# Effect of Acute Moderate-Intensity Exercise on the Mirror Neuron System: Role of Cardiovascular Fitness Level

**DOI:** 10.3389/fpsyg.2020.00312

**Published:** 2020-02-21

**Authors:** Zebo Xu, Zi-Rong Wang, Jin Li, Min Hu, Ming-Qiang Xiang

**Affiliations:** ^1^Department of Sports and Health, Guangzhou Sport University, Guangzhou, China; ^2^Department of Linguistics and Modern Languages, The Chinese University of Hong Kong, Hong Kong, China; ^3^Department of Graduation, Guangzhou Sport University, Guangzhou, China

**Keywords:** mirror neuron system, action understanding, social cognition, cardiovascular fitness level, acute moderate-intensity exercise, fNIRS

## Abstract

**Objectives:**

The aims of this study were to use functional near-infrared spectroscopy (fNIRS) to determine whether cardiovascular fitness levels modulate the activation of the mirror neuron system (MNS) under table-setting tasks in non-exercise situation, to replicate the study that positive effect of acute moderate-intensity exercise on the MNS and investigate whether cardiovascular fitness levels modulates the effect of exercise on the activation of the MNS.

**Methods:**

Thirty-six healthy college-aged participants completed a maximal graded exercise test (GXT) and were categorized as high, moderate, or low cardiovascular fitness. Participants then performed table-setting tasks including an action execution task (EXEC) and action observation task (OBS) prior to (PRE) and after (POST) either a rest condition (CTRL) or a cycling exercise condition (EXP). The EXP condition consisted of a 5-min warm-up, 15-min moderate-intensity exercise (65% VO_2__max_), and 5-min cool-down.

**Results:**

No significant differences were observed for Oxy-Hb and Deoxy-Hb between different cardiovascular fitness levels in the EXEC or OBS tasks in the non-exercise session. But there were significant improvements of oxygenated hemoglobin (Oxy-Hb) in the inferior frontal gyrus (IFG) and pre-motor area (PMC) regions under the OBS task following the acute moderate exercise. Particularly, the improvements (Post-Pre) of Δ Oxy-Hb were mainly observed in high and low fitness individuals. There was also a significant improvement of deoxygenated hemoglobin (Deoxy-Hb) in the IPL region under the OBS task. The following analysis indicated that exercise improved Δ Deoxy-Hb in high fitness individuals.

**Conclusion:**

This study indicated that the activation of MNS was not modulated by the cardiovascular fitness levels in the non-exercise situation. We replicated the previous study that moderate exercise improved activation of MNS; we also provided the first empirical evidence that moderate-intensity exercise positively affects the MNS activation in college students of high and low cardiovascular fitness levels.

## Introduction

Mirror neuron system (MNS) was activated when an individual performed action, and observed the same action performed by others (Sun et al., 2018). The first discovery of MNS was in the ventral premotor cortex (area F5) of the macaque brain; it fired when grasping food as well as when the macaque observed the experimenter grasping food. Then, the MNS was found in the rostral inferior parietal lobule (IPL) (PF/PFG), also firing when a monkey executes a goal-related action and mouth actions, as well as observing the same action in another subject (Rizzolatti et al., 1996; Gallese et al., 2004; Fogassi et al., 2005). Previous work has determined the location of the MNS in the human (Buccino et al., 2001; Gallese et al., 2004; Filimon et al., 2007; Kilner et al., 2009; Molenberghs et al., 2010) and its functions for action understanding (Johnson-Frey et al., 2003; Leslie et al., 2004) and imitation (Buccino et al., 2004; Bernier et al., 2013). A more general hypothesis was that the MNS also played a crucial role in social cognition to catch the intentions and emotions of others (Gallese, 2006; Pfeifer et al., 2008; Perkins et al., 2010). Then Language evolved became a powerful and flexible tool when humans developed a social function to exchange knowledge (Tomasello et al., 2005). However, several studies were skeptical about the role that the MNS played in social cognition, arguing that the MNS was simply the motor controller and did not include action understanding which is one of the most important basic functions in social cognition (Baird et al., 2011; Hickok et al., 2011b). Besides, other studies declared that the dorsal part of the premotor cortex in MNS did play a role in action understanding, but only the dorsomedial prefrontal of MNS which was called the mentalizing system (MENT) activated by the social relevant tasks (Spunt and Lieberman, 2012; Geiger et al., 2019).

In the field of sport psychology, cardiovascular fitness level was considered as one of the most important factors. Several studies have indicated that high fitness was associated with greater brain volume and functional connectivity (Chaddock et al., 2010; Voss et al., 2010). The role of fitness in the cognitive performance was also investigated in prior studies. Åberg et al. (2009) have shown that young adulthood with higher fitness levels would perform better in cognitive tasks. And one study researched on 877 older adults indicated that higher fitness level was associated with better motor skills, cognitive performance, and memory (Freudenberger et al., 2016). Although several studies revealed higher fitness levels related to better daily performances, there is still no study to reveal the relationship between cardiovascular fitness levels and the activation of MNS in the action understanding tasks which might be the basic neural mechanism to social function, language function, and cognitive function.

Exercise has also been shown to benefit cognition (Audiffren et al., 2009; Byun et al., 2014), the hippocampus and memory (Sayal, 2015), and improved motor control in early Parkinson’s disease patients (Fisher et al., 2008), social behaviors in children with autism (Bremer et al., 2016), as well as adolescents with attention-deficit/hyperactivity disorder (ADHD) (Kamp et al., 2014). Previous work done by Drollette et al. (2012) has shown that preadolescent children had a greater performance on the cognitive control task (Flanker task) after 15 min of moderate-intensity running at 60% maximal heart rate (HR_max_) compared with resting state. Furthermore, a recent study used functional near-infrared spectroscopy (fNIRS) also demonstrated that moderate-intensity exercise could improve the activation of MNS in an action understanding social task (Xu et al., 2019), which indirectly outlined one of the neural bases of exercise improved social behaviors in children with autism. Although Xu et al. (2019) have shown the positive effect of exercise on the MNS, more studies are still needed to verify this effect.

With in-depth study, some previous studies have observed different effects of exercise on cognitive performance among different cardiovascular fitness levels. For example, Chu et al. (2015) found that acute moderate-intensity exercise can improve the performance of cognitive functions and to a specific improvement in the executive function of high and low cardiovascular fitness levels in older adults. Chang et al. (2012) also indicated the improvement of cognitive performances which are the information processing, attention, and executive function tasks after a delay of light and moderate-intensity exercise on high and low-fit younger adults in their meta-analysis study. Previous reviews also highly recommended cardiovascular fitness should be measured and analyzed in the study (Brisswalter et al., 2002; Tomporowski, 2003). Those studies mentioned above indicated that cardiovascular fitness might modulate the effect of exercise on cognitive functions, since the function of MNS was relevant with cognitive control, for example, patients with impairment of motor control and aphasia (mouth action control) after stroke were also following less activation of MNS (Small et al., 2012). However, how cardiovascular fitness level modulates the effects of acute exercise on the MNS in action understanding tasks remains less understood and warrants more explorations.

Therefore, the aims of this study were to determine whether MNS activation is related to the cardiovascular fitness level in the non-exercise situation, to replicate a study done by Xu et al. (2019), that is acute exercise can improve MNS response in the action understanding tasks and to evaluate whether the effect of moderate-intensity exercise on MNS is modulated by cardiovascular fitness, Specifically, we also made the following hypotheses.

Hypothesis 1: Because prior studies illustrated that higher cardiovascular fitness level was related to better cognitive performance (Freudenberger et al., 2016), the MNS regions with high fitness individuals should exhibit the largest activation compare with moderate and low fitness groups in non-exercise session under our action understanding tasks.

Hypothesis 2: Because the previous study has shown moderate-intensity exercise increased activation of MNS in the OBS task (Xu et al., 2019), thus, parts of MNS regions activation will be increased after exercise in the OBS task.

Hypothesis 3: Because plenty of evidence indicated that the effect of exercise mainly benefits high-and low-fit individuals’ cognitive performances (Chang et al., 2012), the subgroup-analyses will show the improvements of MNS in cardiovascular high and low fitness level individuals following exercise under action understanding tasks.

## Materials And Methods

### Participants

Thirty-six college-aged participants were recruited to this study (mean age 20.6 ± 1.5 years, height 169 ± 9 cm, body, weight 61.4 ± 12.5 kg; 16 females). All participants were healthy and right-handed (Edinburgh Handedness Inventory score > 0.85), and all had a normal or corrected-to-normal vision. All participants completed four sessions (body test, experimental, control, and acute exercise sessions) and were instructed to avoid any intense exercise in 24 h between each session. All participants completed a maximal oxygen consumption test and were then split into three groups based on the American College of Sports Medicine (ACSM) guidelines (American College of Sports Medicine [ACSM], 2013). The maximal oxygen consumption (VO_2__max_) of each fitness group was categorized as: low fitness group, moderate fitness group, and high fitness group (Table 1). According to ACSM guidelines, these groups have previously been described as having poor (35.4–43.5 ml/kg/min for male; 26.2–33.6 ml/kg/min for female), fair (43.5–49.1 ml/kg/min for male; 33.6–38.9 ml/kg/min for female), and good fitness (49.1 above ml/kg/min for male; 38.9 above ml/kg/min for female), respectively (American College of Sports Medicine [ACSM], 2013). Written informed consent was obtained from all participants in accordance with the Declaration of Helsinki. The protocol was approved by the Ethics Committee of Guangzhou Sport University.

**TABLE 1:** Participants’ demographic and physiological characteristics for low, middle, and high fitness groups (mean ± SD).

Variable	High fitness	Moderate fitness	Low fitness	Total
Sample size	12	13	11	36
Gender (male)	8	7	5	20
Age (yr)	20.44 ± 1.62	20.93 ± 1.49	20.27 ± 1.27	20.58 ± 1.48
Height (cm)	171.05 ± 6.38	168.87 ± 9.54	167.64 ± 10.54	168.53 ± 8.70
Weight (kg)	62.59 ± 9.81	63.71 ± 11.92	60.06 ± 15.624	61.44 ± 12.53
BMI (kg.m^–2^)	21.27 ± 2.09	22.19 ± 2.80	21.07 ± 3.48	21.45 ± 2.92
IPAQ (METs/wk)	3342 ± 1726	3909 ± 2501	2125 ± 1286	2960 ± 1863
VO_2__max_ (mL.kg^–1^.min^–1^) for women	41.78 ± 1.68^a^	36.83 ± 0.69^b^	32.37 ± 1.65^c^	36.43 ± 3.94
VO_2__max_ (mL.kg^–1^.min^–1^) for men	55.74 ± 3.02^a^	48.01 ± 1.26^b^	41.56 ± 2.72^c^	49.49 ± 6.26
VO_2__max_ (mL.kg^–1^.min^–1^) for men and women	51.08 ± 1.80^a^	42.85 ± 1.74^b^	36.60 ± 1.88^c^	43.69 ± 8.44

*BMI, body mass index; IPAQ, International Physical Activity Questionnaire; MET, metabolic equivalent; means with different superscripts a, b, and c are significantly different from one another.*

### Experimental Procedures

In the first session, participants were fully informed regarding each experimental session. Each participant gave written informed consent and filled out an International Physical Activity Questionnaire (IPAQ). Participants meeting the inclusion criteria then performed a test of cardiovascular fitness to VO_2__max_ and were categorized into high, moderate, and low fitness group according to the ACSM guidelines (American College of Sports Medicine [ACSM], 2013).

**FIGURE 1:**
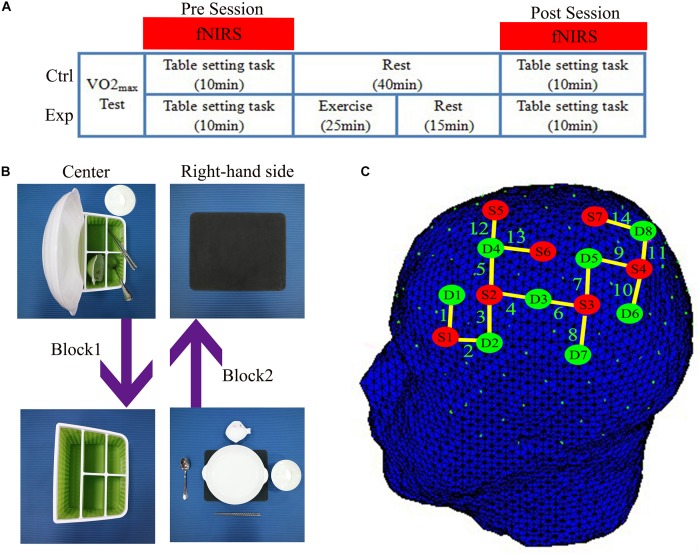
**(A)** The experimental design showed the experimental (Exp) condition and the control (Ctrl) condition. Using functional near-infrared spectroscopy (fNIRS) to measure cortical hemodynamic changes while subjects performed the table-setting tasks before (Pre) and after (Post) exercise or rest. **(B)** Illustration of the design of the table-setting tasks. **(C)** fNIRS optode placement according to the 10–10 International System. Red dots represent the position of light sources. Green dots represent the position of detectors. Yellow lines depict channels.

The second and third sessions were the table-setting task, which has both action execution (EXEC) and action observations (OBS) tasks under experimental (EXP) and control (CTRL) conditions. By definition, acute exercise session occurred only in the EXP condition. During the acute exercise session, all participants performed 25 min of exercise on a cycle ergometer (Ergoselect 100, ergoline GmbH, Germany) that consisted of a 5 min warm-up, 15 min of exercise at moderate intensity (65% HR_max_), and a 5 min recovery period. Heart rate (HR) was monitored by a wireless HR monitor (Acentas pulse meter, BM-CS5EU, Beijing, China). The initial cycling workload was 30 W and automatically increased in the warm-up period until HR reached 65% HR_max._ The cycle ergometer system automatically adjusts the workload if the HR is higher than target HR to ensure they were exercising at moderate intensity over the 15-min exercise period. Finally, participants were allowed to cool down during the recovery period at 30 W. Under the CTRL condition, participants conducted the same action execution and observation components, but rested instead of performing the exercise (Figure 1A).

All participants performed the table-setting task before (PRE) and after (POST) the acute exercise session or rest in the EXP and CTRL conditions. If participants individually attended exercise sessions of the experiment firstly, they performed another session on different days.

### Maximal Oxygen Consumption Test

All participants had their body mass index (BMI) measured in the laboratory. The IPAQ was used to assess participants’ physical activity. Cardiovascular fitness was measured using cardiopulmonary exercise testing (Jaeger-Masterscreen-CPX, Carefusion, Germany). All participants ran on a treadmill (h/p/cosmos airwalk, Germany) using the Bruce protocol for the maximal graded exercise test (GXT) (Bruce et al., 1973). VO_2__max_ was determined if participants met at least three of the following four criteria: (1) respiratory exchange rate (RER) ≥ 1.15; (2) volitional exhaustion; (3) no increase in HR with increasing intensity; (4) rating of perceived exertion (RPE) ≥ 17 (Borg, 1982; Seifert et al., 2010). Participants were asked to rate their exertion on the RPE scale in the last 20 s of the GXT intensity stage before increasing workload.

### The Table-Setting Task to Reflect the MNS Activity

In the initial period, participants and the experimenter (the experimenter is male in this study) sat face to face. The storage box was placed in front of the participant and the placemat placed on their right-hand side. The storage box included five table items: a plate, a saucer, a pair of chopsticks, a soupspoon, and a rice bowl. A monitor placed at a 45° angle in front of participants to presented visual cues.

In the executive (EXEC) task of the experiment, the participants were instructed to place the table items orderly onto and round the placemat with a normal, natural speed, and rhythm in 15 s after the cue was on the monitor: a picture of a cup. Participants were instructed to only use their right-hand and avoid any other movements. Then, their eyes continued to focus on the monitor that displays a cross to remind participants to remain still and avoid any movements for 20 s; this is block 1. In block 2, the four table items will be restored and placed into the storage box, with the bowl placed in front of the box in the identical order in 15 s, then with 20 s to rest. There were eight blocks in this task, block 3 and 4, block 5 and 6, block 7 and 8 were the same as block 1 and 2. The order of placement was always fixed: plate, saucer, chopsticks, soup spoon, and rice bowl (Figure 1B).

In the observation (OBS) task of the experiment, the table items, storage box, and placemat were turned toward to the experimenter. The visual procedures were the same as the EXEC task. The experimenter moved the table items, and participants carefully observed the movements. When the experimenter rested and watched the cross on the monitor, participants also focused on the cross and avoided any movements during the whole OBS task.

### fNIRS Data Acquisition

fNIRS data were acquired using the NIRSport system (NIRx Medical Technologies, LLC, Glen Head, NY, United States). Probe-channel sets were installed with reference to the international 10/10 system into a NIRS-EEG compatible cap (EASYCAP, Herrsching, Germany) and then placed on the participant’s head. The cap position was centered at the Cz point, and then thin plastic straps were inserted between probes to ensure that the distance was less than 3 cm between each source and detector. The fNIRS system consisted of eight light sources and eight detectors which formed 14 channels covering most of the MNS region on the participant’s left hemisphere. Channels 1, 2, and 3 consisted of IFG (BA44/45), channels 4, 5, 12, and 13 consisted of PMC (BA6), channels 6, 7, and 8 consisted of rostral IPL (BA40), and channels 9, 10, 11, and 14 consisted of SPL (BA7) (Figure 1C). An already existing NIRS 10 × 10 positions were used to estimate the NMI coordinates of optodes with respect to the EEG 10/5 positions. The locations of NIRS channels were defined using the maximum probability method (Table 2). The ROIs were determined while three or four channels covered one Brodmann Area. We placed channels only in the left hemisphere because the left hemisphere is dominant when subjects perform a right-handed action (Filimon et al., 2007; Egetemeir et al., 2011). Prior to recording, the NIRStar acquisition software (NIRx Medical Technologies, LLC, Glen Head, NY, United States) recorded fNIRS data and verified the signal quality according to the NIRStar manual. The baseline was set while each participant was resting for 15 s prior to the table-setting test to remove irrelevant noise and signal drift (Fu et al., 2016).

**TABLE 2:** The MNI coordinate of each channel, the source, and detector positions are in the 10-10 system.

		MNI coordinate			
Channel	Source—Detector	*X*	*Y*	*Z*	Brodmann area and anatomical label (percentage overlap)
1	F5–F3	−46	39	26	45—Pars triangularis Broca’s area (72.56%)
2	F5–FC5	−56	24	20	45—Pars triangularis Broca’s area (53.08%)
3	FC3–FC5	−55	12	34	44—Pars opercularis, part of Broca’s area (47.81%)
4	FC3–C3	−50	−3	50	6—Pre-motor and supplementary motor cortex (61.71%)
5	FC3–FC1	−38	12	55	6—Pre-motor and supplementary motor cortex (37.52%)
6	CP3–C3	−52	−34	52	40—Supramarginal gyrus part of Wernicke’s area (43.32%)
7	CP3–CP1	−39	−48	60	40—Supramarginal gyrus part of Wernicke’s area (41.82%)
8	CP3–CP5	−57	−48	38	40—Supramarginal gyrus part of Wernicke’s area (65.46%)
9	P1–CP1	−24	−62	62	7—Somatosensory association cortex (82.72%)
10	P1–P3	−32	−73	47	7—Somatosensory association cortex (69.67%)
11	P1–PZ	−13	−73	56	7—Somatosensory association cortex (91.59%)
12	FCZ–FC1	−13	12	67	6—Pre-motor and supplementary motor cortex (73.21%)
13	C1–FC1	−26	−5	68	6—Pre-motor and supplementary motor cortex (81.78%)
14	CPZ–PZ	2	−61	66	7—Somatosensory association cortex (58.83%)

*The Brodmann area with maximum probability was used in final location of each channel.*

### Data Preprocessing

Raw data from each participant were processed within the nirsLAB analysis package (v2017.06, NIRx Medical Technologies, LLC, Los Angeles, CA, United States). Discontinuities were automatically corrected or deleted by the nirsLAB (std threshold = 5). Spikes were interpolated or manually deleted. A bandpass filter was used; 0.01 Hz was used to remove drift and 0.1 Hz was used to filter respiratory noise. We used the modified Beer–Lambert law (Cope et al., 1988) to analyze the optical data from the fNIRS system. The changes in oxygenated hemoglobin (Oxy-Hb), deoxygenated hemoglobin (Deoxy-Hb), and total hemoglobin (Total-Hb) concentration data were collected at a sampling rate set at 7.81 Hz.

### Statistical Analyses

All descriptive characteristics (age, height, weight, BMI, IPAQ, and VO_2__max_) were imported into IBM SPSS Statistics 22 (SPSS Inc., Chicago, IL, United States), and then a one-way ANOVA was used to compared characteristics between cardiovascular fitness levels (high, moderate, and low).

The statistical parametric mapping (SPM) level 1 (within-subject) package incorporated into nirsLAB was based on the canonical hemodynamic function (parameters in nirsLAB = [6 16 1 1 6 0 32]) to determine event-related changes in Oxy-Hb, Deoxy-Hb, and total-Hb during action execution and observation. Finally, the *beta* values of the Oxy-Hb and Deoxy-Hb were exported from each participant for statistical analysis. Second-level analyses (SPM 2) level 2 assessed differences in groups to export brain activation maps.

The *beta* values of the Oxy-Hb and Deoxy-Hb from each ROI of the participant as the dependent variable were imported to IBM SPSS Statistics 22 (SPSS Inc., Chicago, IL, United States). The one-way ANOVA was applied to compare Oxy-Hb and Deoxy-Hb between levels in non-exercise session (CTRL PRE, CTRL POST, and EXP PRE sessions). Then Oxy-Hb and Deoxy-Hb were subjected to three-way repeated measure ANOVA under different tasks (EXEC and OBS) with three factors: conditions (EXP and CTRL), time sessions (PRE and POST) as within-subject factors, cardiovascular fitness levels (high, moderate, and low) as a between-subject factor. Then when it exhibited main effect on conditions and three-way interaction was significantly different, following two-way repeated measure ANOVA used Bonferroni correction method was applied to the [Post–Pre] Oxy-Hb or Deoxy-Hb contrast (Δ Oxy-Hb and Δ Deoxy-Hb) of EXP and CTRL conditions in these ROIs to compare the effect of exercise on the MNS-related regions that were known as activated by action execution and action observation. We used contrast value of Oxy-Hb or Deoxy-Hb because it would help to eliminate potential variations as different MNS activation may be caused by doing the tasks on different days. All values are presented as mean ± SE. An alpha of 0.05 was used as the statistical significance level for all comparisons.

## Results

### Participant Characteristics

We summarize the basic descriptive characteristics for the three-fitness level. One-way ANOVA indicated no significant difference among fitness levels on the demographic variables of age, height, weight, BMI, and IPAQ. As expected, VO_2__max_ was significantly different between fitness levels [*F*(2, 33) = 15.73, *P* < 0.001], and *post hoc* analyses revealed that all three groups were significantly different from each other. The high fitness level group showed the highest VO_2__max_ value, the moderate fitness group followed, and the low fitness group had the lowest value (Table 1).

### Cortical Hemodynamic Change in OBS Task

In the OBS task, the one-way ANOVA was conducted on each ROI to determine whether the Oxy-Hb and Deoxy-Hb were significant differences between levels in non-exercise session (CTRL PRE, CTRL POST, and EXP PRE sessions). However, it revealed no significant differences between cardiovascular fitness levels with regard to Oxy-Hb and Deoxy-Hb.

**FIGURE 2:**
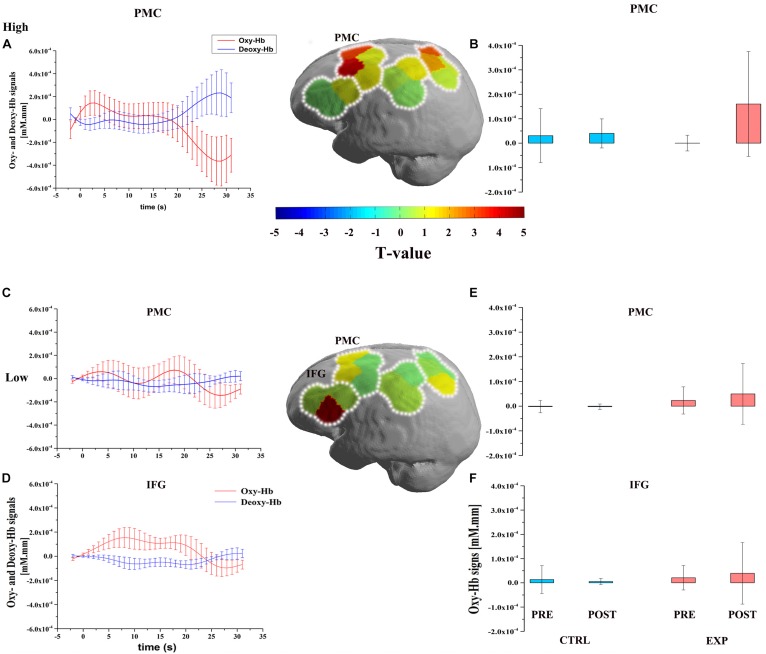
Figures describe the significant improvement of oxygenated hemoglobin Δ Oxy-Hb response after exercise during the OBS task. The upper part is the cortical activation patterns with high cardiovascular fitness level (high: **A,B**) and the lower part is the cortical activation patterns with low cardiovascular fitness level (low: **C–F**). Three graphs of first column depict the timelines for the significant Δ Oxy-Hb (red) and Δ Deoxy-Hb (blue) signal which are shown in arbitrary units (mM⋅mm) **(A,C,D)**. Significant Oxy-Hb signal changes in ROIs during the OBS task in all conditions are shown in the last column. Error bars indicate standard error **(B,E,F)**. Middle figures are t-map of oxy-Hb signal contrast activation in the OBS task. T-values are shown according to the color bar.

The three-way repeated measures ANOVA was performed on each of the ROI of the Oxy-Hb and Deoxy-Hb. It revealed a significant main effect on conditions [*F*(2,33) = 9.30, *P* < 0.05, η^2^ = 0.22], a significant interaction between conditions, time sessions, and cardiovascular fitness levels [*F*(2,33) = 3.42, *P* < 0.05, η*^2^* = 0.17] in IFG region. It also revealed a significant main effect on conditions [*F*(2,33) = 4.70, *P* < 0.05, η*^2^* = 0.13] and a marginal significant interaction [*F*(2,33) = 2.70, *P* = 0.08, η*^2^* = 0.14] in the PMC region. However, there was no significant interaction in the IPL and SPL regions. In order to determine which fitness level of activation of these two ROIs were increased by exercise, two-way repeated measure ANOVA was used to compare the contrast value (Δ Oxy-Hb) of EXP and CTRL conditions. It indicated that was a significant difference in the low fitness level [*F*(2,33) = 5.11, *P* < 0.05, η*^2^* = 0.13, Bonferroni-corrected] in IFG region. Also, it exhibited significant difference in the high fitness level [*F*(2,33) = 17.36, *P* < 0.001, η*^2^* = 0.35, Bonferroni-corrected] and low fitness level [*F*(2,33) = 7.62, *P* < 0.05, η*^2^* = 0.19, Bonferroni-corrected] in the PMC region. However, there was no significant difference in PMC and IFG regions in the moderate fitness level (Figure 2).

With regard to Deoxy-Hb, there was a significant main effect in conditions [*F*(2,33) = 7.92, *P* < 0.05, η*^2^* = 0.19], a significant interaction between conditions, time sessions, and cardiovascular fitness levels [*F*(2,33) = 3.33, *P* < 0.05, η*^2^* = 0.17] in IPL region. In order to determine which fitness level of activation of this ROI was increased by exercise, two-way repeated measure ANOVA was used to compare the contrast value (Δ Deoxy-Hb) of EXP and CTRL conditions. It indicated that was a significant difference in the high fitness level [*F*(2,33) = 6.14, *P* < 0.05, η*^2^* = 0.16, Bonferroni-corrected] in IPL region. There was no significant difference in the IPL region in the moderate and low fitness levels.

### Cortical Hemodynamic Change in EXEC Task

In the EXEC condition, the one-way ANOVA was also conducted to each ROI determine whether the Oxy-Hb and Deoxy-Hb were significantly different between levels of fitness in the non-exercise session, which were not affected by exercise. The results revealed no significant differences between cardiovascular fitness levels for Oxy-Hb and Deoxy-Hb.

The three-way repeated measures ANOVA was also performed on each of the ROI of Oxy-Hb and Deoxy-Hb, it revealed no significant main effect on conditions or interaction between conditions, time sessions, and cardiovascular fitness levels for both Oxy-Hb and Deoxy-Hb. The data that support the findings of this study are openly available in Mendeley at http://dx.doi.org/10.17632/s8tp7d75dw.1.

## Discussion

Our study indicated that there was no significant difference between fitness levels with EXEC or OBS task under non-exercise session, which implied different cardiovascular fitness levels could not reflect different activation of MNS under this table-setting social task and denied Hypothesis 1. Wright et al. (2019) evidenced that physical development is positively correlated with cognitive performance, language ability, and social-emotional state. Higher fitness level has been shown that was associated with better average accuracy and response time across all level of spatial memory tasks, lower switch cost in elderly adults, these higher fitness older adults also showed a greater functional connectivity which was related to better cognitive function (Voss et al., 2010; Prakash et al., 2011). Some investigations have shown that physical fitness level was relevant to language development. One study indicated that compared with typical developmental children, children with developmental language disorders showed worse performance on vertical jump (Muursepp et al., 2014). Also, the physical fitness performance of children with developmental language disorders was significantly lower than those of typical children (van der Niet et al., 2014). Although our study showed that there was no significant difference between cardiovascular fitness levels and MNS activation, it does not conflict with previous works because the cognitive function, social cognition, and language function are still different functions in the human brain, and participants were both college students which generally excluded language developmental disorders. And the most crucial point is that the values of VO_2__max_ in the low level of our participants were also higher than those with language developmental disorders relatively. More studies are needed to determine whether cardiovascular fitness level reflects social and language cognition.

Mirror neuron system has been reported to be an action execution and observation matching system. It also played a crucial role in the development of motor and language functions (Nishitani and Hari, 2000; Tettamanti et al., 2005). Our study illustrated that moderate-intensity exercise has a positive effect on the functions of MNS by increasing the Oxy-Hb during an OBS task. This result was consistent with Hypothesis 2 and other similar studies, which have shown the beneficial effect of moderate-intensity exercise on executive functions as indexed by the increasing Oxy-Hb (Yanagisawa et al., 2010; Byun et al., 2014). Therefore, the result of this study implied that moderate exercise might be an effective way to improve social cognition by activating the MNS. Our result was consistent with recent studies showed that moderate exercise benefited those who have social cognition deficits social behaviors such as autism spectrum disorder (ASD) (Magnusson et al., 2012; Schmitz et al., 2017), in whom an MNS deficit has been described (Theoret et al., 2005; Hadjikhani et al., 2006). fMRI studies have also suggested no mirror neuron activity in the inferior frontal gyrus (pars opercularis in children with autism during imitation of emotional expressions (Dapretto et al., 2005), as well as during observation of human motion (Martineau et al., 2010). Our study indirectly provided one of the first pieces of neural basic evidence that exercise can improve the social behaviors of children with autism by improving the activation of MNS.

In addition, the subgroup analysis also showed that moderate exercise benefited people with high and low fitness levels, but not at moderate fitness level. Thus, Hypothesis 3 was supported. Specifically, it revealed the improvement of MNS activation in high fitness levels of the PMC region and improved the MNS activation of low fitness level individuals in IFG and PMC regions under OBS task. AS we can find plenty evidence to support acute exercise improved human brain functions of high and low fit individuals, recent studies have demonstrated the positive effect of acute exercise on cognitive performance of high and low fitness group in old adults (Chang et al., 2012, 2015; Chu et al., 2015). For instance, Chu et al. (2015) recruited forty-six healthy older adults to do a reading control and Stroop tasks after 30 min aerobic exercise training, the results revealed that acute exercise improved the performance of these two types of cognitive functions in both high and low fitness level old adults. Moreover, Hogan et al. (2013) demonstrated that unfit group has lower error rates in the flanker task under the exercise condition compared with rest condition and faster RTs were observed in fit participants after exercise. Although a previous meta-analysis has reported that fitness level significantly modulated the positive effects of exercise for low fit and high fit participants after a delay following exercise (Chang et al., 2012), In contrast, Chang et al. (2014) indicated acute exercise can improve performance in the congruent condition of Stroop task in all levels of cardiovascular fitness, but individuals of high cardiovascular fitness level demonstrated longer response times under incongruent condition. More research is needed since particularly in the context that social cognition and executive function are two different brain functions. Possible explanations for this result is the different values of VO_2__max_ were used in different studies to categorized different fitness levels (Chang et al., 2014), the intensity of exercise and possibly different durations of exercise protocol led to different results (Chang et al., 2012). In addition, we hypothesized that social cognition is more sensitive to the stimulation of exercise when individuals are of low cardiovascular fitness, which can be easily aroused in a short period of time. However, individuals of moderate fitness are accustomed to moderate exercise, such that they experience marginal returns on social cognition improvement. Only when individuals reach a high fitness level in a long-term training program, can social cognition enjoy the greatest benefits from exercise since our body is in an optimal condition. There were few studies that have used neuroimaging methods to investigate the effect of exercise on different aerobic fitness levels participants’ cognition (Pesce, 2009). Therefore, more research is required to determine how and why moderate-intensity exercise benefits MNS in high and low fitness individuals.

Besides, PMC area as one of important MNS regions, it showed improvements of activation in both high and low fit individuals under the action understanding task (OBS). This result can be supported by previous study demonstrated the positive effect of exercise intervention on Parkinson’s disease patients, they showed that exercise increased motor control in early Parkinson’s disease patients (Fisher et al., 2008; Shah et al., 2016). In Petzinger et al. (2013)’s review, they summarized exercise intervention to enable the goal-based motor skill training to engage the cognitive circuit to motor learning. Exercise increased the blood flow and facilitated the neuroplasticity in elderly adults, so this has the potential to result in the improvement of both cognitive and automatic components of motor control. Thus, our study indirectly explained why exercise intervention increased the self-perceived capability through instruction and feedback (reinforcement) in Parkinson’s elderly adults, which can be explained by improving the action understanding function after exercise.

We also discuss whether the exercise only improved one of the functions of MNS which is motor control. Or exercise only improved action understanding but not to reach the social cognition function. Hickok et al. (2011b) suggest that MNS was merely a motor control or action selection function, and does not include understanding action performing by oneself or others. They provided much evidence to show that information flowing down into the temporal lobes was used to connect our visual and auditory experience with memories of conceptual objects and the dorsal stream processes that same visual information to integrate with the PMC region to generate movement. As the MNS was part of this dorsal stream, the output of motor (Hickok et al., 2011a). However, previous studies also indicated perform social tasks could activate PMC regions (Buccino et al., 2001; Molnar-Szakacs et al., 2006; Perkins et al., 2015), this evidence supported MNS function involves action understanding to social recognition. Moreover, our study implied that the MNS basic function is not only a motor controller since participants remained still and simply observed the action will not requiring any extra motor control function in this process (OBS tasks), the result indicated improvements in Oxy-Hb and Deoxy-Hb in action observation task after exercise to support moderate exercise was indeed stimulating the action understanding function. Similar to the argument mentioned above, some researchers also argued that parts of MNS (dorsal part of PMC) was responded by the action understanding, but the theory of mind or social cognition function is control by MENT (dorsal part of mPFC and the IFG as another part of MNS) (Geiger et al., 2019), even this statement is true, the result from our study indicated that exercise might benefit social cognition is still valid. Since in IFG region, the activation of low-level participants was still improved.

## Limitation and Future Research

The present study might be limited because the Oxy-Hb and Deoxy-Hb values within each fitness level between men and women were not categorized identically, this issue due to the limit number of participants in each fitness level. Therefore, these issues may limit the generalizability of our findings and future research should identify the role of sex on exercise and the MNS. However, since the factor of gender has already been counter-balanced in this study (four out of 12 were female in high fitness, six out of 13 were female in moderate, and six out of 11 were female in low fitness), we can assume that effect of gender would not bias the results.

Another question is whether we should use measures of Oxy-Hb or Deoxy-Hb to represent behavioral data. Some studies have indicated that the Oxy-Hb signal is often observed to have a higher amplitude than the Deoxy-Hb signal (Strangman et al., 2002; Yanagisawa et al., 2010), which means that Oxy-Hb is more sensitive to the task response (Cheng et al., 2015). Our study also illustrated more amplitude changes using Oxy-Hb signal compared with Deoxy-Hb. However, we were still able to observe some trace when using Deoxy-Hb in our study, which suggests that Deoxy-Hb data from fNIRS is still necessary to include in the analysis for a more comprehensive picture.

As our study only used the acute aerobic exercise protocol, and different protocols of exercise have indicated different effects on cognitive performances, expanded researches into effect of long-term exercise, and different exercise protocols of training programs are necessary to determine the effect of exercise on MNS. And more task protocols involve behavioral index associate with social cognition performance should be included into neuroimaging studies since we only used the Oxy-Hb and Deoxy-Hb as the index to indicate the effect of exercise on the MNS.

## Data Availability Statement

All datasets generated for this study are included in the article/supplementary material.

## Ethics Statement

The studies involving human participants were reviewed and approved by the Ethics Committee of Guangzhou Sport University. The patients/participants provided their written informed consent to participate in this study. Written informed consent was obtained from the individual(s) for the publication of any potentially identifiable images or data included in this article.

## Author Contributions

ZX, MH, and M-QX contributed to conception and design of the study. ZX, Z-RW, and JL organized the database. ZX and M-QX analyzed the data. ZX wrote the first draft of the manuscript. MH and M-QX contributed to manuscript revision and read and approved the submitted version.

## Conflict of Interest

The authors declare that the research was conducted in the absence of any commercial or financial relationships that could be construed as a potential conflict of interest.
